# Perineal protection techniques during labor

**DOI:** 10.18332/ejm/190068

**Published:** 2024-07-05

**Authors:** Eszter Borján

**Affiliations:** 1 Department of Clinical Studies in Obstetrics and Gynaecology, Faculty of Health Sciences, Semmelweis University, Budapest, Hungary

**Keywords:** midwifery practice, perineal laceration, perineal massage, manual perineal protection

**Dear Editor,**

Giving birth is one of the most important events in a woman’s life^1,2^. Human evolution led to some anatomical modifications, which could cause difficulties in childbirth. The bipedal position meant a series of physical adaptations in the body that provided certain advantages; however, it caused problems at the time of delivery due to the narrowing of the pelvic canal, which made it difficult for the fetal head to pass^1-3^. The human fetus emerges from the birth canal facing in the opposite direction from its mother, so it is difficult to reach down, as non-human primate mothers often do. The adaptation that humans have made to this situation is to need assistance during deliveries^2,3^.

Every year, more than 130 million babies are born worldwide^4^. It is reported that approximately 85% of women having a vaginal birth sustain some degree of perineal trauma^4^. Genital tract traumas are classified into four subtypes according to the location and severity/depth of the lesion. Third- and fourth-degree tears are collectively called obstetric anal sphincter injuries: OASIS^4-6^.

The perineal trauma is associated with short- and long-term morbidity^5,6^. Perineal damage may result in dyspareunia, urinary and fecal incontinence, bowel dysfunction, infections, hemorrhage, persistent perineal pain, weakness of the pelvic floor musculature, uterine prolapse, cystocele, and rectocele^3,7^.

Numerous techniques worldwide have been practiced by midwives and obstetricians with the aim of reducing trauma during delivery; although some are applied in the antenatal period, most are used during the second stage of labor^4,8^. Nowadays, it is still a controversial issue; some defend active protection of the perineum (manual perineal support) or ‘hands-on’, and others instead support expectant protection of the perineum, also known as ‘hands-off’ or hands-poised^5,6,8,9^.

The effectiveness of episiotomy is not clear; due to the fact that some believe it is protective^5,6^, others suggest it makes little or no difference^6^.

Many articles analyze perineal massage in the second stage of labor. Some authors suggest that it reduces the degree of the laceration^5,6,8^; others suggest avoiding this measure until further research can prove its benefits^6,8,10^.

Reducing the incidence of perineal trauma should be one of the main challenges of birth attendants. The prevention of perineal trauma is a combination of many techniques; moreover, there is controversy when recommending one technique or another. Meta-analyses did not demonstrate a statistically significant protective effect of manual perineal support on the risk of OA-SIS^5,6,7,11^. In our practice, we use a hands-on technique: the manual protection of the perineum during delivery of the head and hand on the infant’s head to slow down the speed of the delivery (extension of the head) (Figure 1).

**Figure 1:**
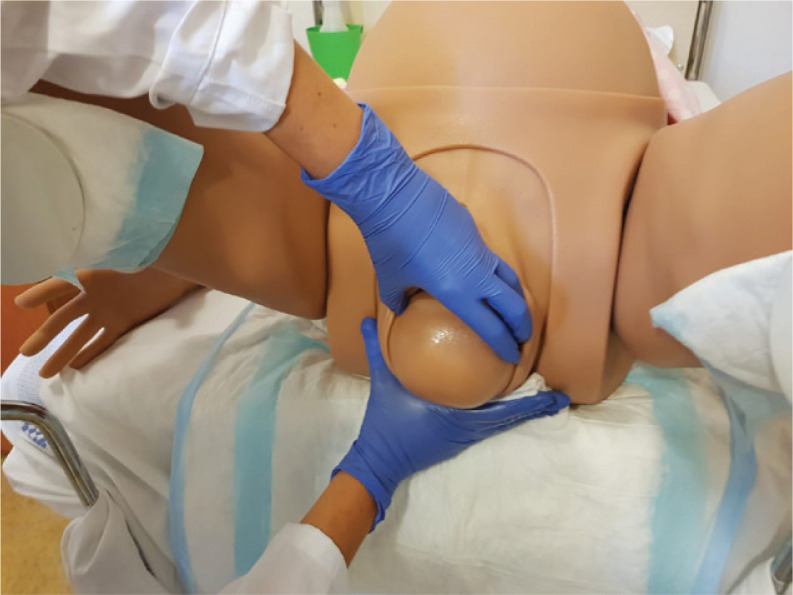
Example of perineal protection technique

We believe that manual perineal support should not be examined in isolation, but we should consider many other factors that could influence the risk of laceration: parity, size of the fetal head, rapidity of labor, position of mother, flexibility of the perineum, and adequate communication among the mother and birth attendants. Birth attendants are required to consider the findings of systematic reviews and meta-analyses in this area and adjust their daily practices and protocols accordingly.

## Data Availability

Data sharing is not applicable to this article as no new data were created.
